# Supplementary material for: Atomistic characterization of the active-site solvation dynamics of a model photocatalyst
Source: Nat Commun. 2016 Nov 28;7:13678. doi: 10.1038/ncomms13678 (PMC5133712; doi:10.1038/ncomms13678)
Supplement: Supplementary Information — Supplementary Figures 1-16, Supplementary Methods and Supplementary References. [file ncomms13678-s1.pdf]

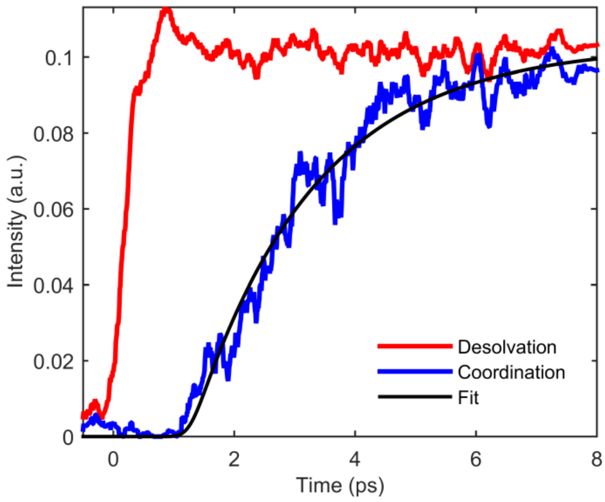

Supplementary Figure 1 | Extended Solvent Dynamics of photoexited $[Ir_2(dimen)_4]^{2+}$ in acetonitrile.

The experimentally derived solvation response in an extended time range, with the coordination part of the solvation response fitted with a single exponential grow-in broadened by a 130 fs Gaussian to account for the IRF. The exponential fit delivers a delay in the onset of 1.3 ps and a time constant of the grow-in of 2.0 ps.

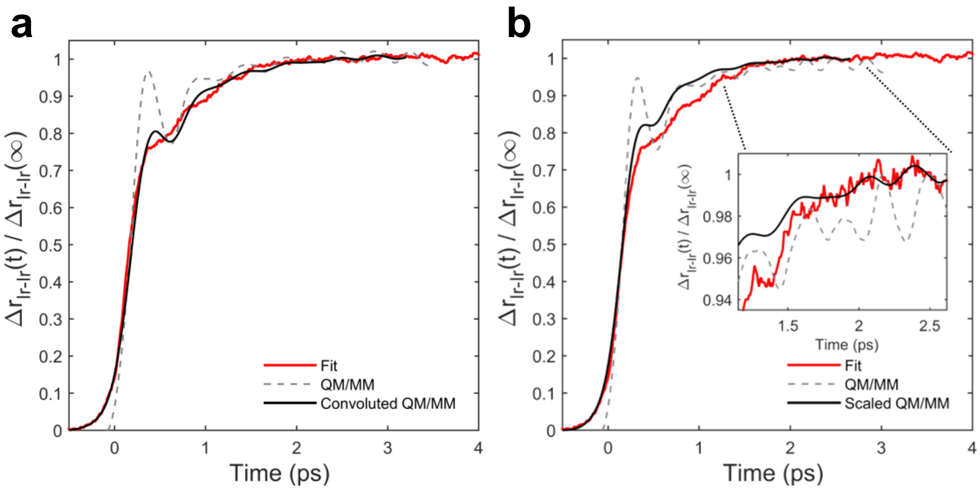

Supplementary Figure 2 | Measured Ir-Ir contraction compared with BOMD simulations.

Comparison of the relative Ir-Ir contraction derived from the analysis of the experimental data (red line) and from the BOMD simulations (black/dashed lines). Left Panel shows a direct comparison between Ir-Ir contraction after rescaling the initial Ir-Ir distance in the BOMD simulations to 4.3Å (from the original 4.6 Å). Right Panel shows a direct comparison between Ir-Ir contraction of experiment and simulation after multiplying the time-axis of the simulation by 0.9 (effectively speeding up the simulation by 10%). This lines up the oscillatory features of experiment and simulation, consistent with the ~7% slower dynamics of the BOMD Ir-Ir vibration compared to previously published optical data[1].

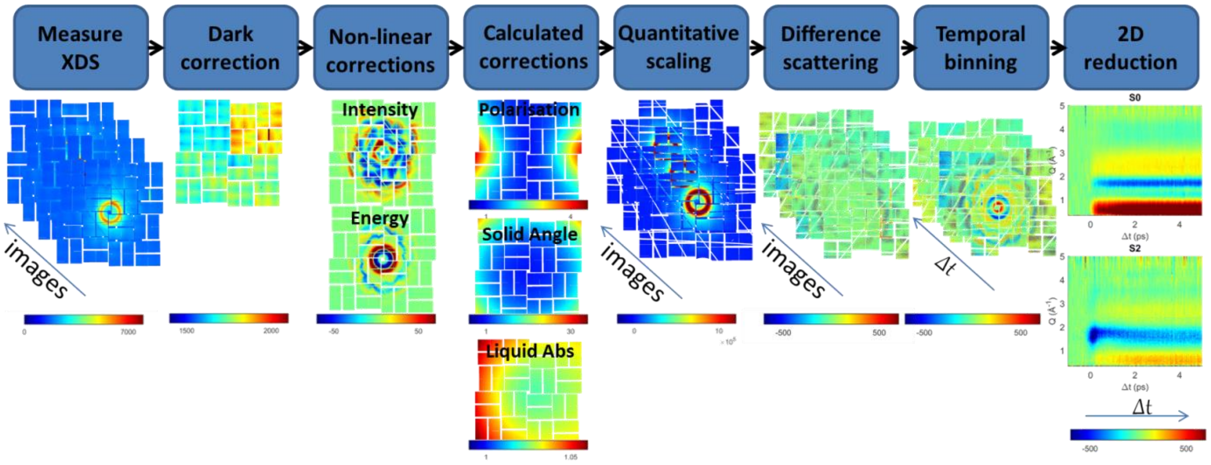

Supplementary Figure 3 | Illustration of the data extraction and reduction procedure. The reduction process consists of multiple consecutive steps which reduces several terabytes of raw 2D scattering data to MB-sized sets of 1D difference scattering curves as a function of time.

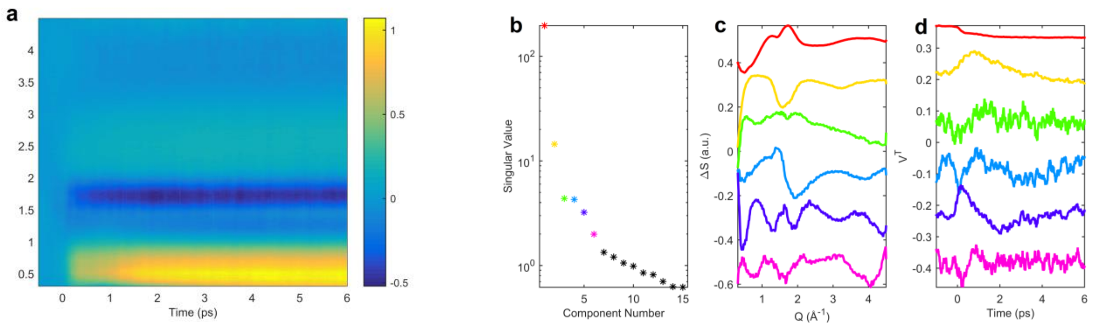

Supplementary Figure 4 | Singular value decomposition of the data. a, shows a 2D plot of the difference scattering data, panel **b-d**, shows the results of the singular value decomposition. **b,** shows the singular values as obtained by the analysis with the first six being highlighted by colors. **c,** shows the corresponding left singular vectors (U_i) describing the signal shape and **d,** shows the right singular vectors (V^T_i) describing the time evolution.

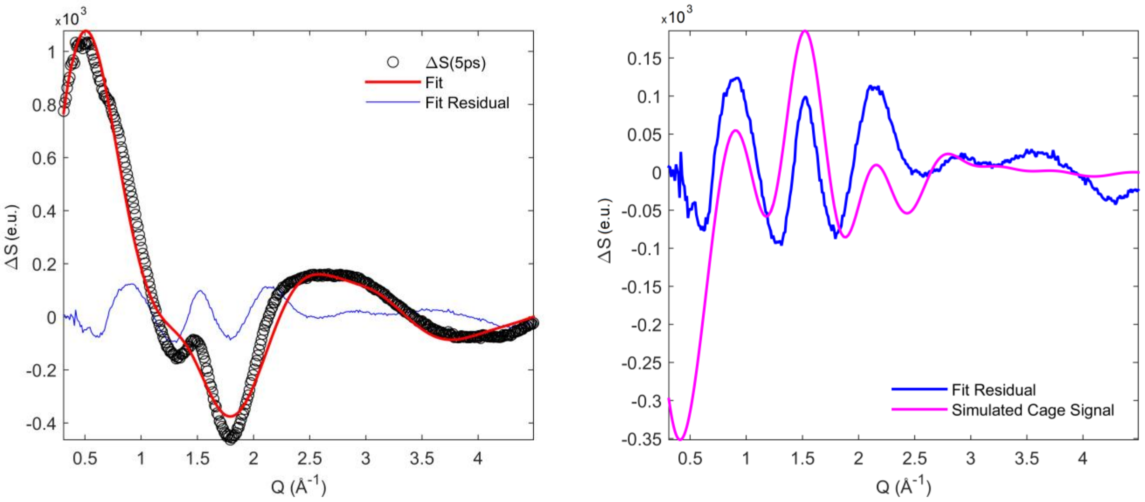

Supplementary Figure 5 | 5ps XDS fit and residual. a, Data, fit and residual of a structural analysis of the $Ir_2(dimen)_4^{2+}$ XDS data at 5 ps, and not accounting for the solvent cage term. **b,** Comparison between the residual and the simulated difference signal of the solute-solvent cage dynamics of the BOMD trajectories.

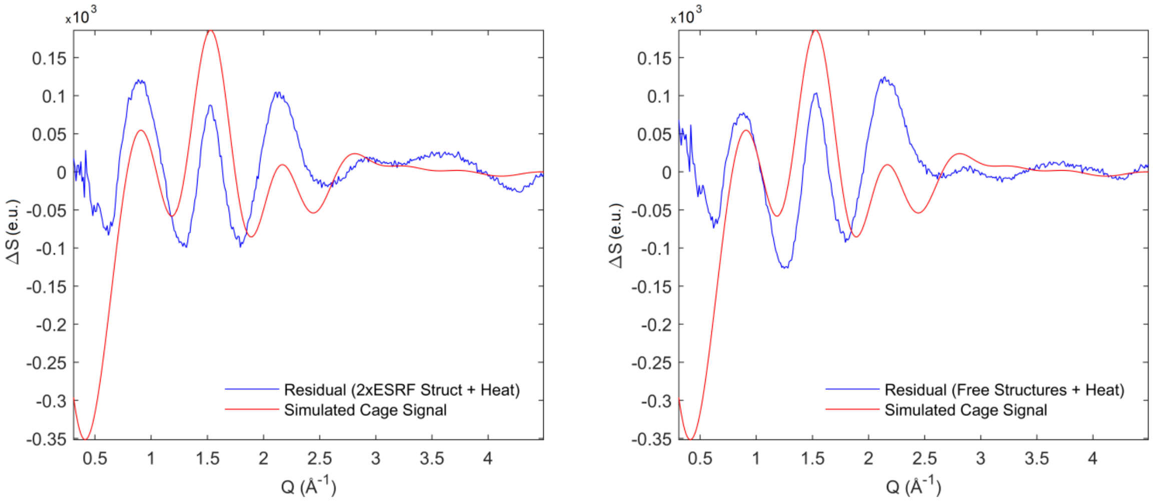

Supplementary Figure 6 | Fit residuals and simulated solvation signal. a, Residual of a structural analysis of the $Ir_2(dimen)_4^{2+}$ data compared to, **b**, the signal simulated from the solute-solvent cage dynamics of the MD trajectories. For **a**, the residual signal is extracted after "locking" the excited state structures to those determined from ESRF data [10], in **b**, the residual signal is extracted after a free structural optimization.

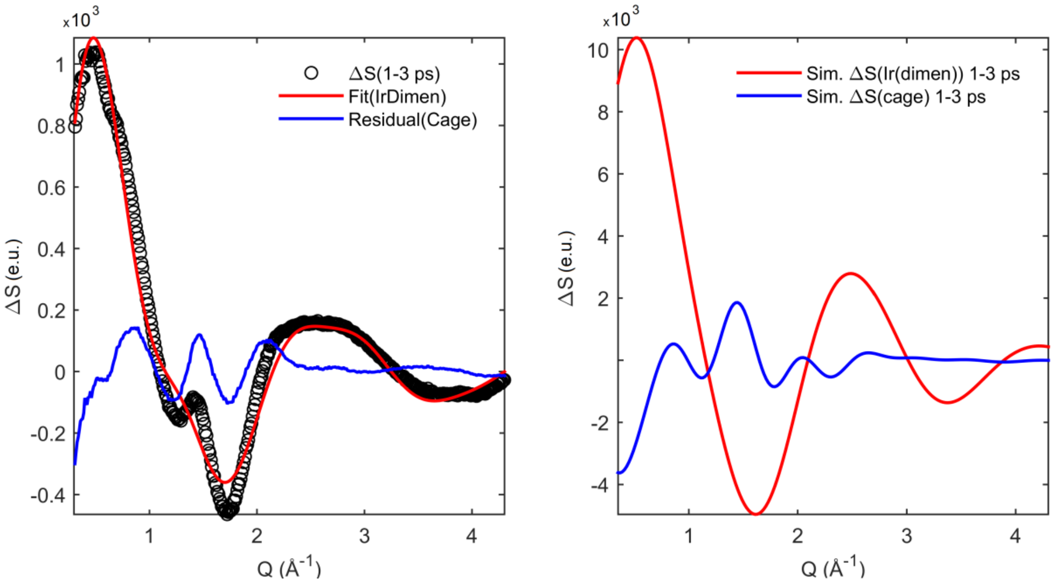

Supplementary Figure 7 | Solute and cage signals from XDS compared with BOMD simulations. **a**, Solute and cage components extracted from the difference scattering data and, **b**, Solute and cage components calculated from the BOMD simulations. The comparison shows that the relative amplitude between solute and cage contribution to the signal is comparable between measured and simulated data. Note that the roughly one order of magnitude difference in signal amplitude correspond to the 13% excitation fraction in experiment compared to 100% excitation fraction in the simulation.

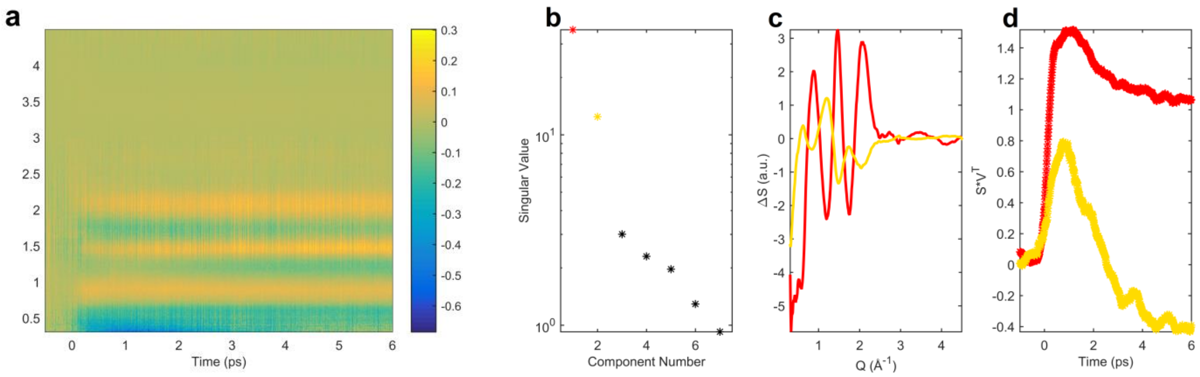

Supplementary Figure 8 | Dynamics of the isolated solvation cage signal. **a,** Residual after subtracting solute components and bulk solvent components from the difference scattering data. **b-d,** Results of a singular value decomposition of the isolated solvation cage signal.

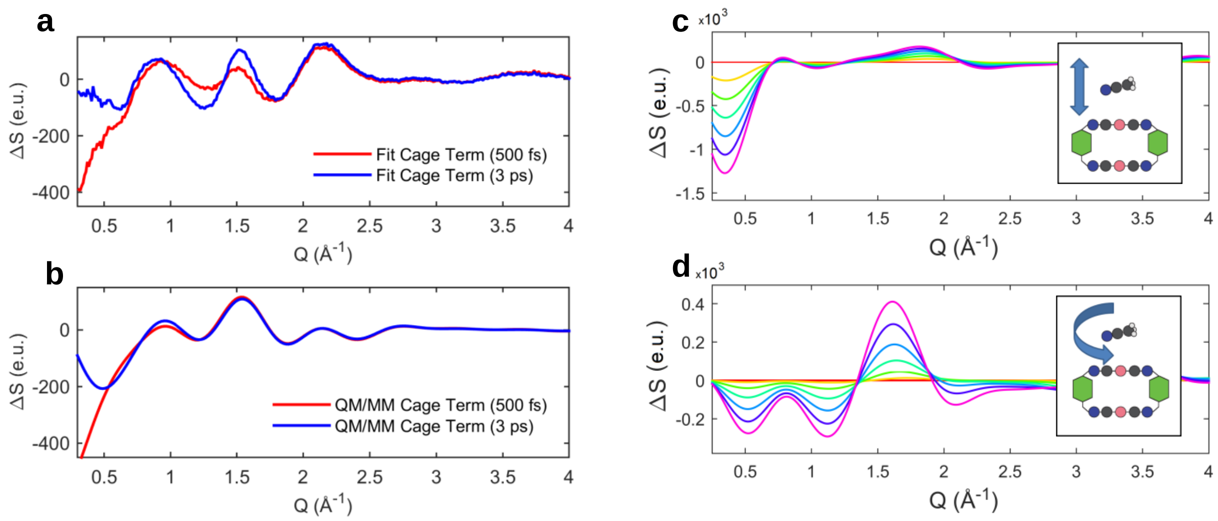

Supplementary Figure 9| Simulated and experimental cage terms compared with a simple model.

a, shows the cage term extracted from the difference scattering data at 500 fs and 3 ps time delay. **b**, shows the solvent cage term simulated from the BOMD at the same time delays, scaled by the 13% excitation fraction. **c** and **d,** simulated signal for the two primary contributions to the solvent cage dynamics. **c**, shows the simulated difference scattering signal when increasing the distance between the $Ir_2(dimen)_4^{2+}$ and an acetontrile molecule placed at 3.6 Å distance along the Ir-Ir axis, and moving the acetonitrile to 4.2 in steps of 0.1 Å. **d**, shows the simulated difference scattering signal when rotating an acetonitrile molecule placed along the Ir-Ir axis at 3.6 Å distance from 90° to 30° with respect to the Ir-Ir axis. Inserts sketch the movements giving rise to the difference scattering signals (gray is carbon, blue is nitrogen, white is hydrogen, pink is Ir and green is the rest of the dimen ligand. The two out-of-plane dimen ligands have been omitted for clarity).

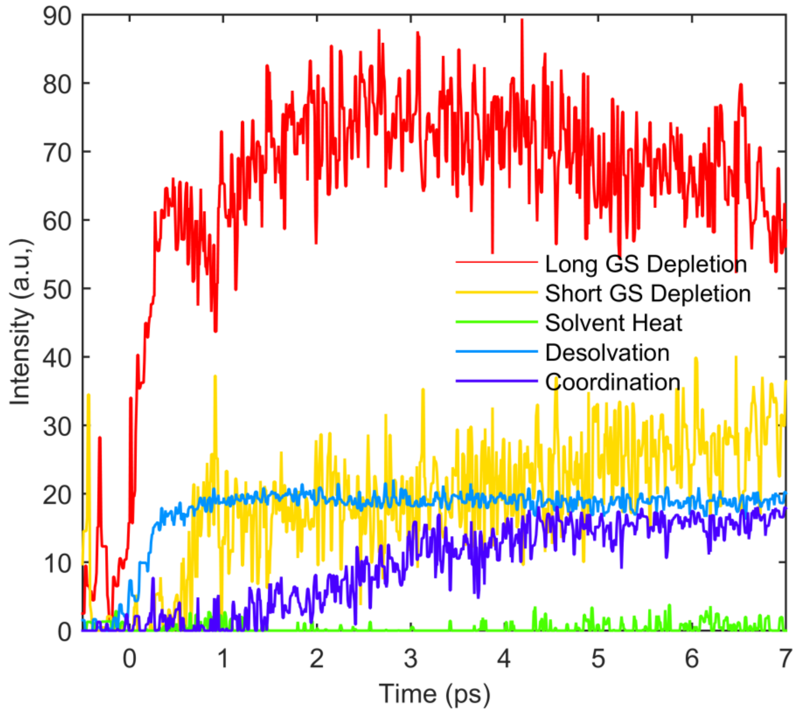

Supplementary Figure 10 | Kinetics of a free 5 parameter fir of the XDS data. The amplitude of the five fit parameters a, b, e, f, g when scaled with their absolute contribution to the fitted difference scattering signal. The amplitudes are the result of an unconstrained fit to the difference scattering data.

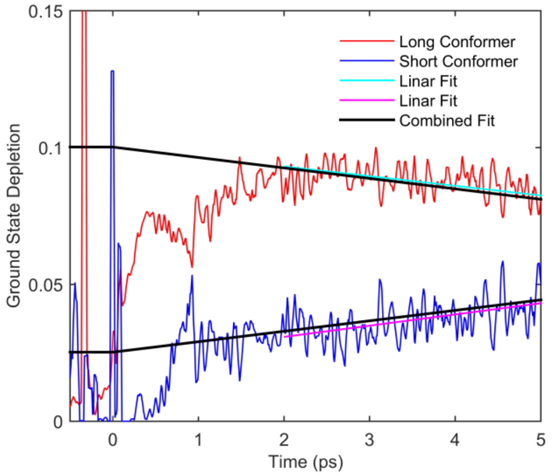

Supplementary Figure 11 | Ground state depletion kinetics. The depletion of each ground state (red, blue), fitted with a linear evolution for time delays t > 2 ps (cyan, magenta). The black lines show a back-extrapolation of the fitted dynamics to t < 2 ps.

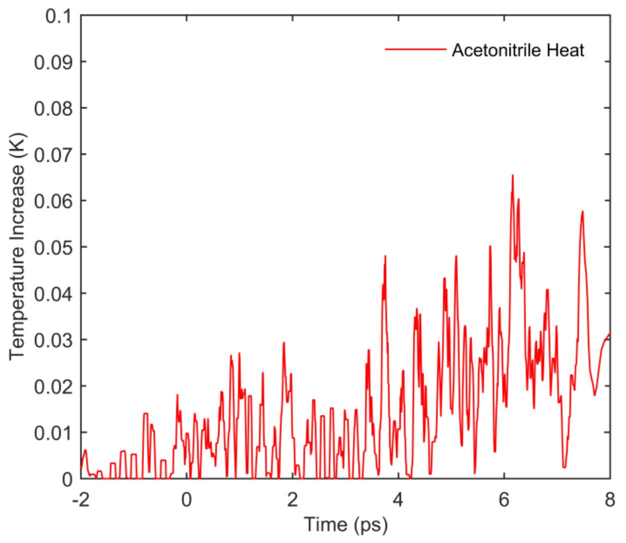

Supplementary Figure 12| Extracted temperature increase of the solvent. The temperature increase derived from the fit of the XDS data showing the temperature increase ΔT(t) in Kelvin.

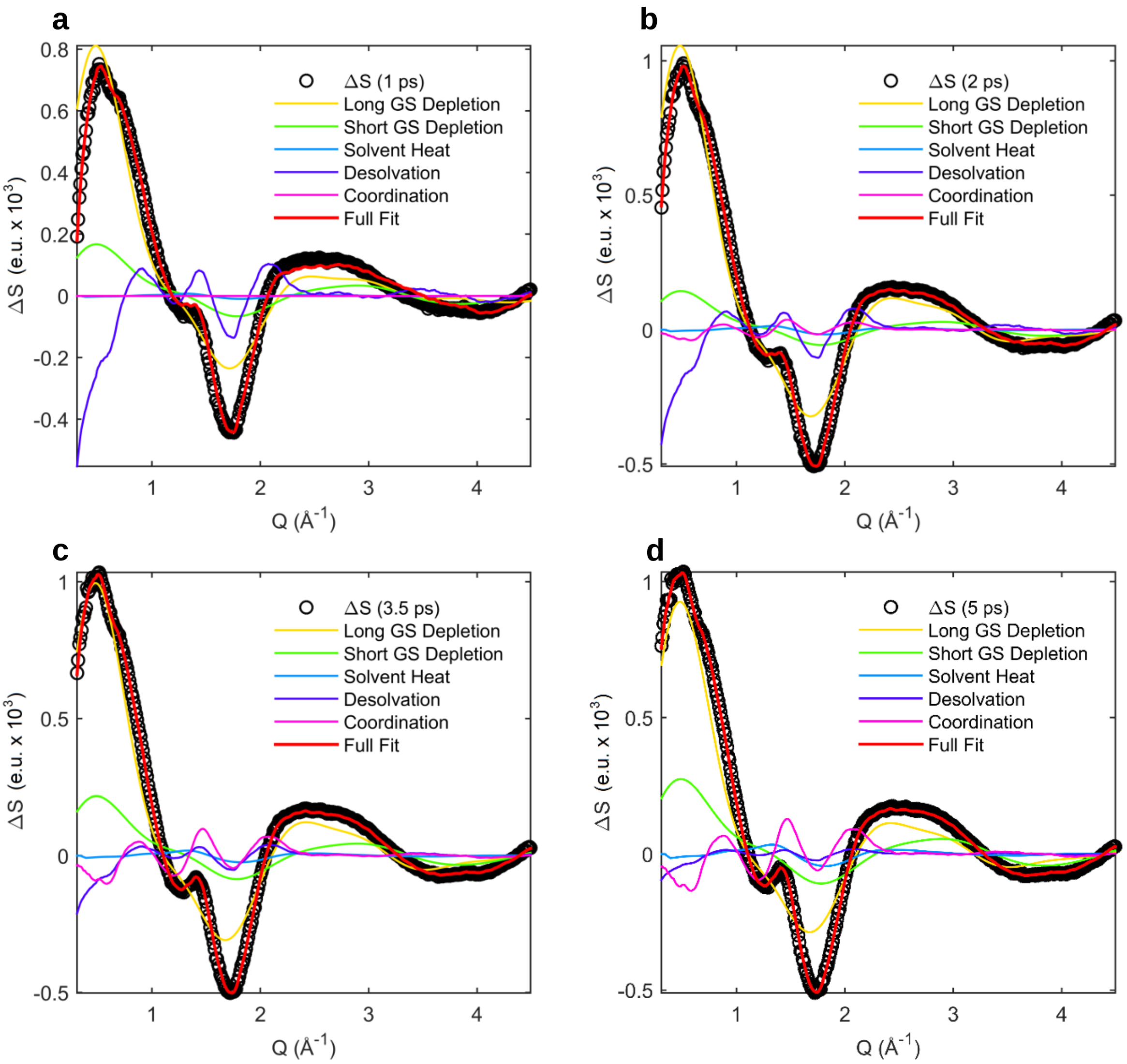

Supplementary Figure 13 | XDS data, fit, and fit components at four different time delays. The fits show the evolution of the contribution from the different fit parameters at **a,** 1 ps, **b**, 2 ps, **c**, 3.5 ps, and **d,** 5 ps time delay.

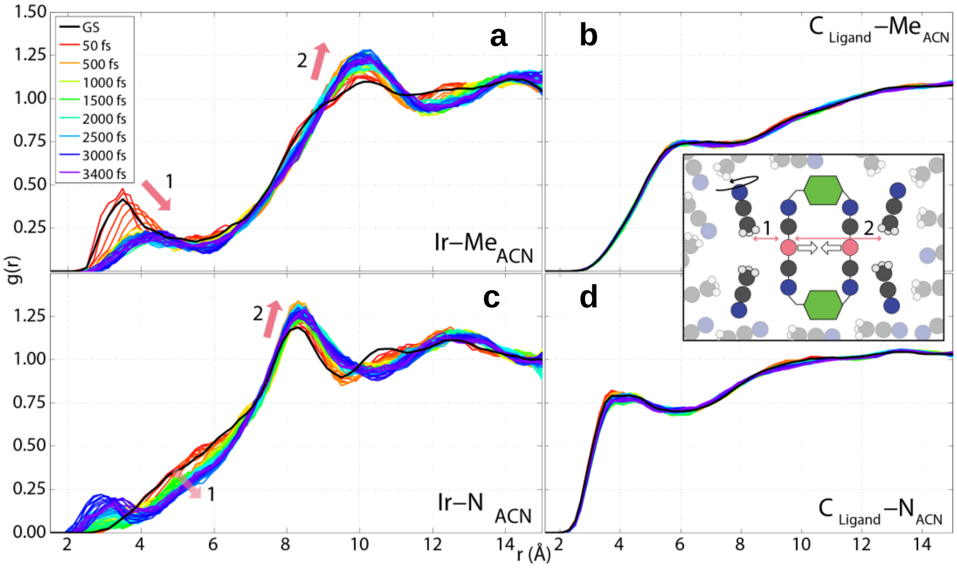

Supplementary Figure 14 | Solvent-related RDFs. **a** and **c,** RDFs between the iridium atoms and each end atom of the solvent molecules. **b** and **d,** RDFs between the dimen ligand carbons and each end of the solvent molecules. The RDFs are sampled with Δr = 0.2 Å at each time step over all the ES trajectories, and temporally averaged with a bin size of 50 fs, and finally smoothed with a 5 point moving average. The arrows shown in the left-hand panels indicate the direction of the time evolution of the PDF at these points, and the interpretation of the dynamic changes is sketched in the cartoon in the inset: The dynamic changes delineated with arrows labeled "1" show a broadening, plus an increase of distance *r*. This is interpreted as the Ir-Ir contraction induced cavity-creation between the Ir atom and the adjacent solvent molecules, i.e. the desolvation. Due to the symmetry of the molecule, the contracting Ir-Ir atoms will also *decrease* their distance to solvent molecules on the *opposite* side of the solute, which is observed at the arrows labeled "2".

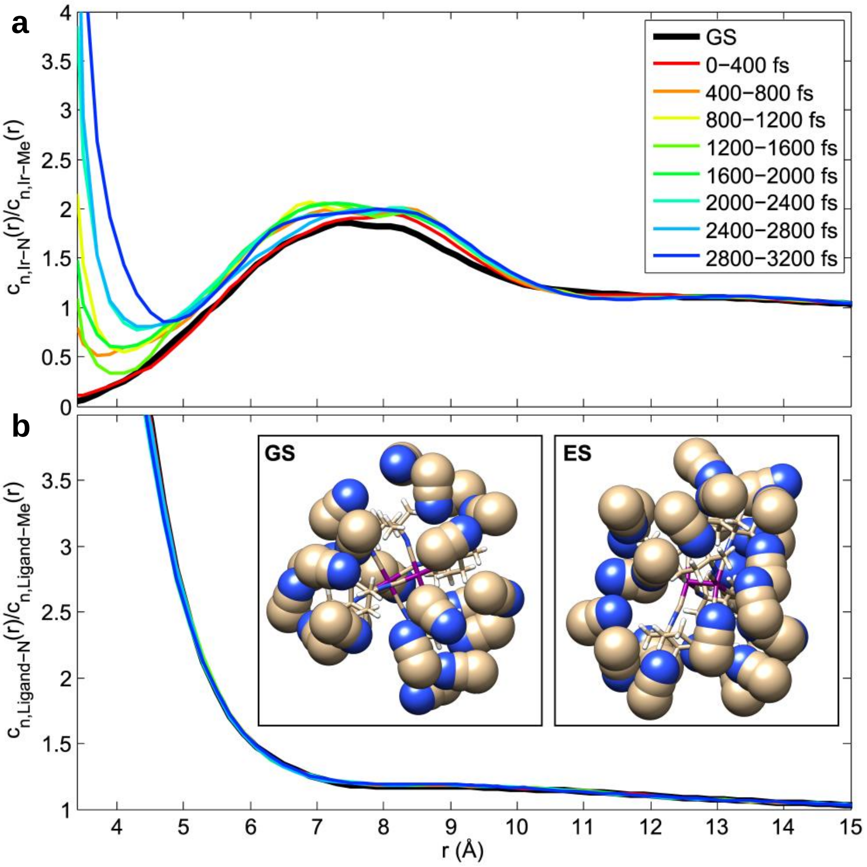

Supplementary Figure 15 | Coordination numbers around the Ir solvation site. a, Coordination number ratios of Ir-N:Ir-Me. **b**, Coordination numbers of Ligand-N:Ligand-Me. Both datasets are averaged in 400 fs bins. For iridium, the GS ratio is below 1 for short distances, but this ratio significantly increases after the first 400 fs. For the ligands, no significant changes are seen, and the ratio is always above 1. In the long-distance limit of the bulk solvent, the ratios converge to 1, which corresponds to the random orientation. The insets in the bottom plot depict snapshots of all the full ACN molecules within 4 Å of the solute. (N = blue, C = gold, H has been omitted for clarity)

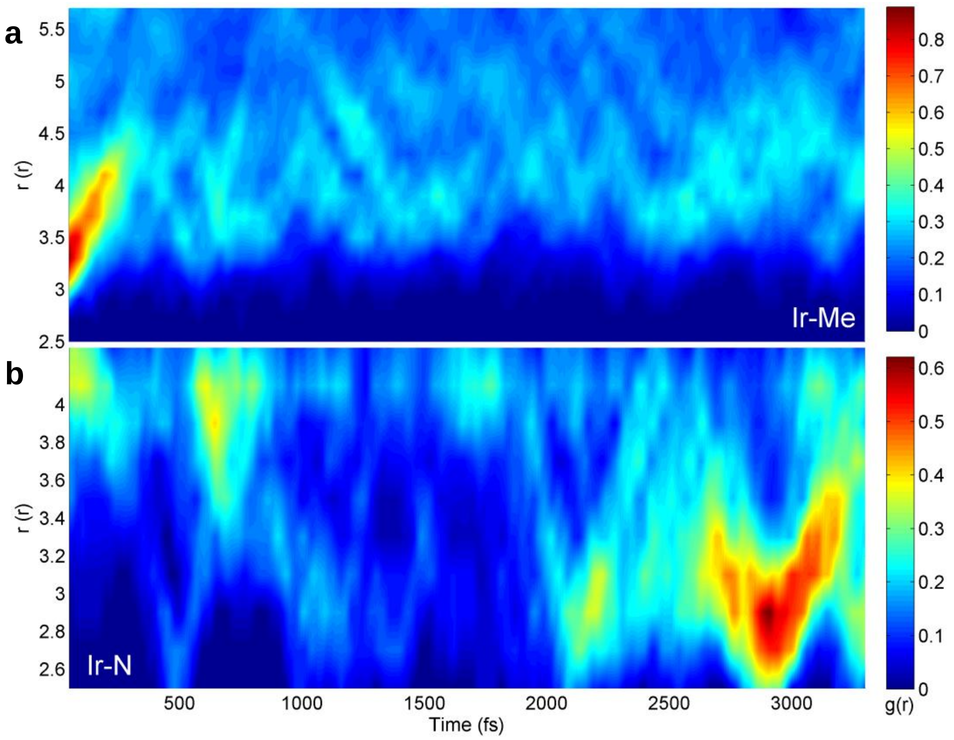

Supplementary Figure 16 | Temporal evolution of the RDFs between Ir and each end of ACN. a, shows the Ir-Me RDF where *r* for the peak increases with time, in conjunction with a decreasing $g(r)$ -value. **b,** shows the Ir-N RDF where the grow-in of a peak at short distances occur after roughly 2 ps.

Supplementary Methods

Data Extraction and Reduction

The XDS data was measured in scans consisting of many individual sets of pump/probe events measured at different time delays. Typically a scan would consist of 30.000 individual images, with 600 images at each nominal time delay being recorded over the course of ~5 seconds. To facilitate the substantial data reduction process described below, every 4th pump/probe event was recorded without laser excitation ("laser drop-shots" or "unpumped" images) and every 5th event was recorded without x-rays ("x-ray drop-shots") resulting in every 20th image with neither the laser excitation pulse nor the x-ray probe pulse arriving at the sample position ("dark images"). Difference scattering images are then constructed by subtracting unpumped images from pumped images. Based on the newly implemented timing tool[2] the data could subsequently be sorted and binned according to actual time delay between x-ray and laser, yielding a data set with a ~130 fs time resolution governed by the combined IRF given by the length of the pump- and probe pulses and the temporal widening due to velocity mismatch in the sample rather than the ~500 fs of the x-ray/laser jitter observed for this set of experiments

The dataflow for the data treatment and reduction is illustrated in Supplementary Figure 3 and is described in detail in the following sections.

Measure XDS: The x-ray diffuse scattering is individually measured for a large number of single X-ray pulses produced by the XFEL at 120Hz. The scattering arising from the interaction between each pulse and the sample is measured on the CSPAD area detector and individually stored. All the diagnostic information about the laser and x-ray pulse along with the corrected pump/probe delay determined by the timing tool is also stored for each pulse to allow sorting and filtering based on the individual shot characteristics and the overall machine/instrument performance on a shot-to-shot basis.

Dark correction: A separate "dark" measurement is conducted where the detector is read out at 120 Hz without incident x-rays for two minutes and averaged to form the "dark image". This allows the electronic background noise of the detector to be determined, and as Supplementary Figure 3 shows, this "dark" contribution to the signal varies significantly across the detector. This dark contribution is observed to be constant over the duration of the measurements, and is subtracted from each image prior to all other corrections. The magnitude of the dark measurement is ~1300 ADU/pixel.

Non-linear corrections: In the second step of the data reduction applied here, the CSPAD images were corrected for X-ray energy- and intensity non-linearity of the individual pixels as described in detail[3]. These signal variations arising from the non-linearity of the detector responses of the same order of magnitude as the difference signals that are the starting point for the data analysis and are therefore removed in order to facilitate the analysis of the difference scattering signals.

The non-linear corrections were determined for each data acquisition scan based on the unpumped images (see above) in each scan, thus avoiding effects from drifts and changes in pulse characteristics and detector response over the time scale of the entire experiment (5 days).

Calculated corrections: Following the corrections for detector nonlinearity, standard detector corrections were applied to every image. These corrections compensate for variations in solid angle coverage by each pixel, for the x-ray polarization as well as for the x-ray absorption through the slightly tilted liquid sheet[4,5]. As a final step in the correction procedure, the 2D images were corrected by binning the individual pixels as a function of 2θ and adjusting a gain factor for each pixels such that a radially isotropic scattering signal of the corrected unpumped XDS data was enforced.

Quantitative scaling: The individual scattering images were put on an absolute scale of electron units/liquid unit-cell by following the 1D scaling-procedure described in detail in previous work[6]. The measured scattering was scaled to a simulated scattering curve at $Q > 3$ Å^{-1}. The simulated scattering curve was calculated from the sum of the coherent and incoherent scattering from an ensemble of molecules representing the stoichiometry of the sample.

Difference scattering : Before calculating the difference signals on which the structural analysis rely, outlier detection and removal was accomplished through a procedure relying on comparing each (azimuthally integrated) image to the median of the rest, a method closely analogous to the one used in our previous work and discussed in detail in the SI of[7], except with the Chauvenet criterion replaced by a median-comparison . Typically ~10% of the images were rejected in this step.

In order to isolate the signal arising from the structural changes induced in the sample by the pump laser pulse, difference scattering images were constructed by subtracting the average of the nearest two (bracketing) unpumped images from each pumped image. This converts the data from each individual acquisition scan to a (large, ~20000 individual images) set of 2D difference scattering images, each of which is “time stamped” with the actual delay time (~10 fs accuracy) between the laser- and X-ray pulse for that particular difference image.

Temporal binning: To improve the S/N-ratio to a level where quantitative analysis of the difference signals is feasible, the data from 26 acquisition scans was combined and sorted according to time delay. This allows an averaging in “temporal bins”, producing averaged difference scattering images in predefined time delay intervals, thus reducing the massive amount of data further significantly increasing the signal to noise ratio of the averaged images.

The data presented here was extracted from a set of 26 consecutive scans comprising ~700.000 individual pump-probe events. To obtain a uniform S/N ratio over the entire time delay range the temporal binning was done for a fixed number of images (200) rather than for a fixed range of time delays. As described above, the data acquisition protocol was designed to ensure almost-uniform temporal coverage in the -2 ps to 4ps region of central interest to these experiments, and the average temporal bin width in this region was approximately 8 femtoseconds

2D data reduction: Due to the short duration of the pulses and short delays studied in these experiments, the difference scattering images contain some anisotropy that will not have had time to rotationally average. Only a subset of solute molecules with a favorable alignment of the transition dipole moment are excited. This preferential excitation results in a population of excited-state molecules where the structural change takes place along the laser polarization . This in turn leads to anisotropic difference scattering signals[8,9]. The anisotropic 2D scattering images can, however, be fully described by two contributions which are a function scattering vector Q only, but which are weighted by the angle with respect to the laser polarization axis. By following the procedure presented in[8], this allows the robust extraction of the isotropic scattering described by the Debye equation and in turn allows all subsequent analysis to be carried out within the general framework applied in our previous work[6,7,10].

Data Analysis

The difference scattering signal - $\Delta S(Q,t)$ - contains contributions from all the structural changes that occur in the probed sample volume following laser excitation. It is usually expressed as the sum of 3 terms:

i) The solute term $\Delta S_{solute}(Q,t)$, arising from structural changes in the solute

ii) The solvent term $\Delta S_{solvent}(Q,t)$, arising from structural changes of the bulk solvent

iii) The solute-solvent cross-term (cage term) $\Delta S_{cage}(Q,t)$, arising from structural changes in the environment local to the solute, such as in the solvent-shell surrounding the solute.

The solute term - $\Delta S_{solute}(Q,t)$

The solute term arise from changes in the scattering signal caused by changes in the solute structure. Since two ground state structures have been identified for $Ir_2(dimen)_4^{2+}$, the solute term needs to account for the depletion of both the depopulated ground states, the structural dynamics of the excited states excited being populated from each of the ground state structures, as well as the fact that the two ground state conformers of $Ir_2(dimen)_4^{2+}$ interconvert on the relevant time scale of the experiment. This can be written as:

$$\Delta S_{solute}(Q,t) = \alpha \begin{bmatrix} \beta_{t=0} \cdot S_{ES1}(Q,t) + (1-\beta_{t=0}) \cdot S_{ES2}(Q,t) - \\ \beta(t) \cdot S_{GS1}(Q) - (1-\beta(t)) \cdot S_{GS2}(Q) \end{bmatrix}$$

Where α is the concentration of excited states and β(t) and (1- β(t)) are the relative fractions of depleted ground state structures. $S_{GS1}(Q)$ and $S_{GS2}(Q)$ are the simulated scattering signals from the two ground state structures, $S_{ES1}(Q,t)$, $S_{ES2}(Q,t)$ are the simulated scattering signals (see below) signal from the excited state population excited from each of the two ground state configurations. In this representation, the time evolution of the difference signal arises from both the excited state structure as well as from the ground state depletion distribution. Thus $S_{ES1,ES2}(Q,t<0) = S_{GS1,GS2}(Q)$,

The scattering signal from the ground and excited state structures ($S_{GS1}(Q)$, $S_{GS2}(Q)$, $S_{ES1}(Q,t)$ and $S_{ES2}(Q,t)$) was calculated from DFT-derived molecular geometries of ground and excited state $Ir_2(dimen)_4^{2+}$, by employing the Debye equation:

$$S(Q) = \sum_k |f_k(Q)|^2 + \sum_{\substack{k,l \\ l\neq k}} f_k(Q) f_l(Q) \cdot \frac{\sin(Qr_{kl})}{Qr_{kl}}$$

Where k, l and m runs over all atoms in the molecule, $f_{k,l}(Q)$ are the atomic form factors, and r_{kl} is the interatomic distances between every atom pair.

The fitting of measured difference scattering signals utilizing sets of such simulated signals from a range of putative molecular geometries is described in detail elsewhere[6], and the computational details of the DFT calculations are described in Section 'Supplementary Methods: Computational Details – DFT'. The Ir-Ir distances of the ground state DFT calculations, were constrained to the Ir-Ir distances identified in[10].

The excited state structures were parameterized in terms of their most significant structural dynamics according to[11]; namely the Ir-Ir distance (d_{Ir-Ir}) and the ligand dihedral twist ($D_{N-Ir-Ir-N}$), i.e. $S_{ES1,ES2}(Q,t) = S_{ES1,ES2}(Q, d_{Ir-Ir}(t), D_{N-Ir-Ir-N}(t))$

For the range of excited state structures used in the analysis, the Ir-Ir distance and ligand dihedral twist were simultaneously constrained in a series of excited-state DFT calculations, such that a matrix of excited state molecular geometries was generated. The Ir-Ir distance was varied between 2.5 Å and 5 Å in steps of 0.5 Å and the ligand dihedral twist was varied between 0 and 50 degrees in steps of 5 degrees. Within the boundaries of this DFT-optimized 'excited state structure matrix' the excited state structure of any combination of Ir-Ir distance and dihedral twist spanned by the matrix could be constructed as a linear interpolation between the nearest structures and from each of these structures the scattering S(Q) could be calculated through the Debye equation introduced above.

The solvent term - $\Delta S_{solvent}(Q,t)$

The solvent term arises from structural changes of the bulk solvent structure associated with changes in the hydrodynamic parameters (temperature, pressure, density) of the solvent. Assuming a classical continuum description, the equilibrated state of the solvent can be expressed as a function of two independent hydrodynamical variables, here chosen as the temperature (T) and the density (ρ). The $\Delta S_{solvent}$ that originates from the bulk-solvent response can be described as a function of their variations $\Delta T(t)$ and $\Delta\rho(t)$. Investigations at free-electron laser and synchrotron sources[12,13,14] have demonstrated that for the laser fluence used in these experiments, a first order treatment is adequate to model the response of the solvent from the few picosecond timescale to the hundreds of milliseconds time scale. Within this framework the solvent term is quantified through the following linear combination:

$$\Delta S_{solvent}(Q,t) = \Delta T(t)\cdot\left.\frac{\partial S(Q,T)}{\partial T}\right|_{\rho} + \Delta\rho(t)\cdot\left.\frac{\partial S(Q,\rho)}{\partial\rho}\right|_{T}$$

where $\left.\frac{\partial S(Q,T)}{\partial T}\right|_{\rho}$ and $\left.\frac{\partial S(Q,\rho)}{\partial\rho}\right|_{T}$ are the difference scattering signals arising from a change in temperature, T, at constant density ,ρ , and from a change in density,ρ , at constant temperature, T, respectively. The reference difference scattering signals, $\left.\frac{\partial S(Q,T)}{\partial T}\right|_{\rho}$ and $\left.\frac{\partial S(Q,\rho)}{\partial\rho}\right|_{T}$ were acquired independently during a dedicated study at ID09b, ESRF[12]. It has been shown that for times t such that $t < d/v_s$, where d is the FWHM of the laser spot and v_s is the speed of sound in the liquid, no thermal expansion of the solvent has yet taken place[14]. With the present experimental conditions (d = 255 µm FWHM and v_s = 1280 m/s for MeCN), thermal expansion is expected to happen on the 200 ns time scale, which is well beyond the temporal window probed in this experiment. Thus, $\Delta S_{solvent}$ reduces to the contribution from impulsive solvent heating:

$$\Delta S_{solvent}(Q,t) = \Delta T(t)\cdot\left.\frac{\partial S(Q)}{\partial T}\right|_{\rho}$$

The validity of ignoring the density response was further verified by trying to include the density term in the fitting procedure. This returned a best-fit value for density change of -0.02 kg/m^3 with an uncertainty of ±0.07 kg/m^3 and we thus conclude any contribution from density changes to be below this 0.001% detection threshold for this parameter.

The cage term - $\Delta S_{cage}(Q,t)$

In the present case of $Ir_2(dimen)_4^{2+}$ the cage term arises predominantly from changes in the structure of the innermost solvent shells of the solute. The Born-Oppenheimer Molecular Dynamics (BOMD) simulations described in the 'Supplementary Methods: Computational Details – BOMD' section shows that two separate processes contribute to the solvation dynamics of $[Ir_2(dimen)_4]^{2+}$ (1) an

initial *desolvation* of acetonitrile methyl groups solvating the axial sites along the Ir-Ir axis of the $Ir_2(dimen)_4^{2+}$ ground state, followed by (2) a *coordination* of these axial sites of the excited state $Ir_2(dimen)_4^{2+}$ by the nitrogens of the acetonitrile solvent molecules. In the following, the difference scattering signal associated with these two processes is referred to as $\Delta S_{desolvation}(Q)$ and $\Delta S_{coordination}(Q)$ respectively. Thus, the cage term can be expressed as:

$$\Delta S_{cage}(Q,t) = a(t)\cdot\Delta S_{desolvation}(Q) + b(t)\cdot\Delta S_{coordination}(Q)$$

Where *a(t)* and *b(t)* are scaling factors.

As described in[15], the cage term can be simulated directly from the BOMD trajectories by simulating the scattering of the pairwise radial distribution functions between solute and solvent atoms (ignoring solvent-solvent and solute-solute atom pairs).

This cage scattering was simulated for both the 20 ps ground state trajectory and for the 40 excited state trajectories (running for 3.5 ps each), and by subtracting simulated ground state cage scattering from that of the excited state, the cage term (difference scattering signal) was simulated. As the desolvation and coordination processes are consecutive, the signal from each process was estimated from the BOMD simulations as the difference scattering signal simulated on early (300 fs) and late (3 ps) time delays respectively (see 'Supplementary Methods: Data Analysis – Data Fitting' for more information).

$\Delta S_{XDS}(Q,t)$ from the sample

Summarizing the previous sections, ΔS can be expressed as:

$$\begin{aligned}\Delta S\ (Q,t) = {} & \alpha\beta_{t=0}\cdot S_{ES1}(Q,\mathbf{d}_{\mathbf{IrIr}},\mathbf{D}_{\mathbf{NIrIrN}}) + \\ & \alpha(1-\beta_{t=0})\cdot S_{ES2}(Q,\mathbf{d}_{\mathbf{IrIr}},\mathbf{D}_{\mathbf{NIrIrN}}) - \\ & \alpha\boldsymbol{\beta}\cdot S_{GS1}(Q) - \alpha(1-\boldsymbol{\beta})\cdot S_{GS2}(Q) + \\ & \mathbf{\Delta T}\cdot\left.\frac{\partial S(Q)}{\partial T}\right|_{\rho} + \mathbf{a}\cdot\Delta S_{desolvation}(Q) + \mathbf{b}\cdot\Delta S_{resolvation}(Q)\end{aligned}$$

where, in this expression, bold face denotes a time-dependent fit parameter. Depletion of the long ground state conformer is given by $\alpha\beta$, depletion of the short ground state conformer is given by $\alpha(1-\beta)$, d_{Ir-Ir} is the excited state Ir-Ir distance, $D_{N-Ir-Ir-N}$ is the excited state ligand twist, ΔT is the acetonitrile temperature increase, $a(t)$ is the desolvation cage term amplitude and $b(t)$ is the coordination cage term amplitude.

The above expression assumes that six distinct time-dependent processes are contributing to the difference signal. A Singular Value Decomposition of the dataset is presented in Supplementary Figure 4. The singular values from the SVD (Supplementary Figure 4b) indicate that six components (colored stars) are necessary to describe the data above the noise-level (black stars), which is described by the magnitude of the singular values decreasing in a linear fashion on a semi-log plot. The corresponding first six singular vectors are presented in Supplementary Figure 4c and their time evolution is presented in Supplementary Figure 4d. The time-evolution of each of the six singular vectors has significant dynamic around time-zero of the experiment. While there is not necessarily a correspondence between singular vectors and physically meaningful components, the

singular value decomposition of the dataset does show that at least six distinct time-resolved processes can be distinguished in the recorded difference scattering data.

Data Fitting

The above equation (**Supplementary Equation 1**) describes the parameterization of the model used to fit the recorded XDS difference signals. The difference signal for each time delay in the acquired data set is fitted independently by optimizing the following seven fit parameters, which are expressed in terms of six time-dependent parameters:

a) Long ground state conformer depletion - $\alpha\beta(t)$
b) Short ground state conformer depletion - $\alpha(1-\beta(t))$
c) Excited state Ir-Ir distance - $d_{Ir-Ir}(t)$
d) Excited state ligand twist - $D_{N-Ir-Ir-N}(t)$
e) Acetonitrile temperature increase - $\Delta T(t)$
f) Desolvation cage term amplitude - $a(t)$
g) Coordination cage term amplitude - $b(t)$

These parameters are assumed to describe the full evolution of the data set. However, even though the acquired set of difference signals hold enough information content for a seven-parameter fit to be feasible[6], it is well known that several of these parameters (In particular the excitation fraction α and the Ir-Ir distance in the excited state) can be strongly correlated and that this affects the robustness and reliability of the fit to a significant degree[6]. We therefore implemented the following five-step fit procedure:

1) Identifying the presence of a cage term in the acquired data.
2) Determine the cage term from the data.
3) Fit the data at all time delays with unconstrained parameters.
4) Use the results of step (3) to quantify the excitation fraction and ground state interconversion on the t>2 ps time scale.
5) Fit the dynamics of the remaining parameters on the t<2 ps time scale using the ground state depletion dynamics determined in step (4).

For all of these steps, the fitting was done using the constrained-minimization fmincon routine implemented in Matlab® with a uniform noise distribution $\sigma(Q)$ estimated from the standard deviation of the 'laser-off' shots.

Step (1) – Showing the need of a solvent cage term in describing the data

To investigate the potential presence of a solute-solvent cage term in the acquired XDS data, the data recorded for time delays around 5 ps (4.7 ps to 5.3 ps) was fitted using parameters (a) through (e) with (c) and (d) (the excited-state structural parameters) locked to the ~100 ps results previously obtained at a synchrotron source[10].

Data, fit and residuals for the 5ps delay are presented in Supplementary Figure 5a. The residual matches closely what we reported for the investigation of excited state $Ir_2(dimen)_4^{2+}$ on longer time scales[10].

Supplementary Figure 5 a, shows the residual of the constrained fit directly compared with the solute-solvent cage term calculated from the BOMD simulations described in the 'Supplementary methods: Computational Details – BOMD' section below. Even though the relative amplitudes of the oscillatory features in the signal vary, good qualitative agreement between the experimental data and the BOMD result is observed. This indicates that a cage response is present in the data and should be accounted for in the analysis.

To ensure that potential bias from possible erroneous structure determination in the earlier synchrotron result is not an issue, another fit was conducted without constraining the excited state structure. Supplementary Figure 6 shows a comparison between the residuals with and without constrained structural parameters and again with a direct comparison with the BOMD cage term. Very little difference between the residual obtained via these two procedures is observed.

Supplementary Figure 7 compares the amplitude of the solute and cage components from the data and the BOMD simulations to ensure that the relative amplitude of the cage component is not mistakenly enhanced in the fit. Supplementary Figure 7a shows the averaged data recorded between 1 and 3 ps (black circles) fitted only with the solute structural components (red curve) and the resulting residual assigned to the cage dynamics (blue curve). Supplementary Figure 7b shows the averaged solute (red) and cage (blue) contribution in the same time frame calculated from the BOMD simulations.

Step (2) – Extraction of the cage signal from the difference scattering data.

As described below in 'Supplementary Methods: Computational Details – BOMD', the BOMD simulations show that the solvation dynamics proceed in two steps, (1) an initial desolvation of acetonitrile methyl groups close to the Ir sites of the $Ir_2(dimen)_4^{2+}$ upon the photoinduced Ir-Ir contraction, followed by (2) an excited state coordination to the Ir sites of the $Ir_2(dimen)_4^{2+}$ by the nitrogen groups of the acetonitrile solvent.

To investigate whether the acquired difference signals contain contributions from such solvation dynamics, the data is fitted with parameters (a) through (e) plus the average of components (f) and (g) calculated from the BOMD simulations. The residual between the data and the fit components (a) through (e) is then considered to be the experimentally extracted cage signal (or residual cage signal). The average BOMD component is included to avoid overfitting the data with solute and solvent-only parameters, and only one (average) BOMD component is used to ensure that we are not inducing dynamics in the experimentally extracted cage signal which are not present in the data. Supplementary Figure 8a shows the extracted cage signal, and Supplementary Figure 8b-d, shows the results of a singular value decomposition of this cage contribution signal. From the amplitude of the singular vectors (Supplementary Figure 8b) it is seen that the first two components are significantly above the background. The corresponding singular vectors are shown in Supplementary Figure 8c, with the first singular vector resembling the simulated cage signal, and the second singular vector being dominated by a strong negative signal at low Q. The time dependent amplitude of these two singular vectors is shown in Supplementary Figure 8d, showing that the first singular vector grow in at time zero and decays slightly, while the second singular vector grows in with a positive amplitude at early time scales and evolves to a negative amplitude at later time scales. This means that the second singular vector contributes with a negative signal at low Q on early time scales, and a positive signal at low Q on late time scales. The need for two singular vectors in describing the solvation cage signal indicates the need for two different components to adequately describe the evolution of this signal.

Supplementary Figure 9a and 9b show the residual cage term at 500 fs and 3 ps time delay directly compared to the BOMD cage terms simulated for same time delays. Again, we observe that the

locations of the main features are reproduced between the extracted and simulated cage response. The primary signal feature observed in the experimentally determined cage contribution which is *not* accounted for in the simulated cage contribution is the increasing amplitude of the oscillatory feature centered at Q=1.4 $Å^{-1}$. To interpret this, we have simulated the difference signal arising from a translation and rotation of an acetonitrile molecule aligned along the Ir-Ir axis of the $Ir_2(dimen)_4^{2+}$. Supplementary Figure 9c and 9d show the primary features of the difference signal arising from a displacement, 9c, and rotation, 9d, inserts show an acetonitrile molecule and a side view of the $Ir_2(dimen)_4^{2+}$ sketching these two different structural changes. For both simulated signals, the starting position of the acetonitrile is the average distance extracted from the BOMD simulations (with the central carbon atom of the acetonitrile placed along the Ir-Ir axis at 3.6 Å from the closest Ir atom). In the displacement simulation, the acetonitrile molecule is moved away by 0.6 Å in steps of 0.1 Å. In the rotation simulation the C-C-N axis of acetonitrile is rotated from 90° to 30° with respect to the Ir-Ir axis. Both the displacement and rotation are sketched in the inserts of Supplementary Figure 9c and d. The difference scattering simulated for the displacement results in the negative feature at low Q which is also experimentally observed, whereas the rotation results in an oscillatory feature centered around 1.4 $Å^{-1}$. Based on these observations, we assign the Q=1.4 $Å^{-1}$ feature in the extracted cage signal to arise from an increased rotational alignment of acetonitrile molecules along the Ir-Ir axis of the excited state $Ir_2(dimen)_4^{2+}$.

In summary the solvation dynamics can be extracted from the difference scattering data, and two components are required to describe them. The experimental cage term agrees well with the simulated cage terms from the BOMD simulations. Comparing the experimental data with a simulation of the difference scattering signal arising from translation and rotation of an acetonitrile molecule aligned along the Ir-Ir axis suggests a larger degree of rotational alignment of the coordinating molecules along the Ir-Ir axis than suggested by the BOMD simulations.

Step (3) – Fitting the difference scattering data set with free parameters.

The data is then fitted utilizing the full 7-parameter model to fit the data. The evolution of the 5 scalar components (components a, b, e, f, g) is shown in Supplementary Figure 10. We here note that both the experimentally determined residual cage terms and the simulated cage terms from the BOMD calculations can be used for the analysis. The structural dynamics derived from the analysis is independent on the choice between residual and BOMD cage terms.

The excited state structural parameters (Ir-Ir distance and ligand dihedral twist), determined for time delays exceeding t=3 ps values are almost identical to the 2.9 Å and 15 degrees of the 100 ps synchrotron measurements[10]. However, the ground state depletion kinetics (red and yellow traces) shows non-physical evolution during the first picoseconds (e.g. the long ground state depletion (red trace) grows during the first few hundred femtoseconds, then decay partially towards the 1 ps time delay, and grows in again slowly over the next 2 ps). We ascribe this non-physical behavior to be caused by a strong correlation between the ground state depletion parameters and the excited state structural parameters. As described in e.g.[10], such strong correlations between parameters can be removed by determining one of them independently and remove it as a free parameter in the remainder of the analysis. In the present case the ground-state depletion dynamics were estimated as described below.

Step (4) - Determining the ground state interconversion on the <2 ps time scale

The ground state kinetics were estimated and constrained by fitting the evolution of the two ground state depletion amplitudes in the time-range exceeding 2 ps where the difference signal exhibits only slowly-varying dynamics, and then back-extrapolating the evolution the into the 0-2 ps region of primary interest to the present study. In this procedure, linear extrapolation was applied. The

ground state depletion kinetics and resulting fit is shown in Supplementary Figure 11 where the ground state depletion kinetics (red and blue trace) are fitted in the t>2 ps region and back-extrapolated to time-zero. The black lines in Supplementary Figure 11 show a linear back-extrapolation where the ground state depletion dynamics were constrained such that there is a 1:1 ratio between loss of the (preferentially excited) long ground state conformer and the grow-in of the short ground state conformer. The ground state depletion dynamics described by the combined fit was used in the following analysis.

The back-extrapolation of the ground-state depletion dynamics makes it possible to quantify the absolute concentration of states excited from each of the two ground state structures $\alpha(\beta(t=0))$ and $\alpha(1-\beta(t=0))$, and we find that immediately after photo-excitation the excited-state fraction was 13%, 10% excited from the long GS conformer and 3% excited from the short GS conformer. This matches the expectations from optical spectroscopy that we should primarily excite the long ground state conformer at 480 nm[1]. For the structural analysis the fit of the time evolution and the back-extrapolation allows us to fix a and b (and thereby $\alpha(t)$ $\beta(t)$) in the analysis to the relationship obtained in the analysis presented above.

Step (5) - Fitting the excited state dynamics with the ground state kinetics locked to the results of step (4)

With the analysis steps described above, the full excited state structural and solvation dynamics can now be determined using Supplementary Equation S.eq.1, but restricting the free parameters of the fit to be: $d_{Ir-Ir}(t)$, $D_{N-Ir-Ir-N}(t)$, $\Delta T(t)$, $a(t)$, and $b(t)$, the dynamics of which are discussed in detail in the main article. The parameters $d_{Ir-Ir}(t)$, $D_{N-Ir-Ir-N}(t)$ for the species excited from either ground state conformer were constrained such that the *relative* structural change towards the final value was the same for the two species. This means that at e.g. ~200 fs time delay when the Ir-Ir contraction is 70% of its final value, the Ir-Ir distance of the ensemble excited from the long ground state conformer (4.3 Å) would have contracted to 3.32 Å, while the Ir-Ir distance of the ensemble excited from the short ground state conformer (3.6 Å) would have contracted to 3.11 Å. By independently fitting $a(t)$, and $b(t)$ and constraining the dynamics of the long and short ground states, the degrees of freedom are limited to physically meaningful ranges. This assumes that the absolute number of excited state molecules that are created by the laser pulse and remains constant within the 5 ps. Constraining the relationship between the structural dynamics of the two ground-state structural imposes the assumption that the excited state potential energy surface is harmonic and that the two ground state conformers are excited to the same excited state potential energy surface. This assumption is valid for the excited state dynamics occurring after the initial Ir-Ir contraction, as illustrated by the wavelength independent Ir-Ir stretch vibration[1]. However, the initial ballistic contraction happening on the <200 fs time scale is assumed to occur along an anharmonic potential energy surface. This entails that the analysis results on the 0-200 fs time scale could be smeared out by the contribution from the 20% of the molecules excited from the short ground state conformer, which might be the reason that the initial Ir-Ir stretch oscillation seems weaker in the extracted experimental kinetics than for the simulation.

The temperature increase of the bulk solvent is not discussed in the main paper, but is shown in Supplementary Figure 12. The contribution to the signal from the acetonitrile temperature increase is observed to be quite small <0.1 K, and accounts for less than 4% of the total difference signal. This puts this observable at the limit of the resolution of our experiment. This is also seen in the ‘free parameter’ fit (Supplementary Figure 10) where the solvent heat is responsible for the smallest contribution.

The relatively low heat influx (0.04 K temperature increase at 6 ps time delay) match well with the expected temperature increase from the 13% excitation fraction of a 6 mM $Ir_2(dimen)_4^{2+}$ solution. The upper bound for the excess excitation energy is given by the 0.81 eV energy difference between the 480 nm excitation and 700 nm emission from the S_1 state[1]. With a volumetric heat capacity of 63.56 J/(mol *K) for acetonitrile[12], complete vibrational cooling of all excited S_1 states should lead to a temperature increase of 0.050 K

The difference signal, fit and fit components at four different time delays are presented in Supplementary Figure 13.

Computational Details – DFT

The scattering from the structures of ground- and excited state $Ir_2(dimen)_4^{2+}$ was simulated via the Debye-expression[15], utilizing DFT-optimized molecular geometries. These were calculated using the ORCA 2.8 program package[16] using the one parameter hybrid version of the Perdew-Burke-Erzerhoff functional with 25% HF exchange, PBE0[17], and Ahlrichs type TZVP basis set[18]. The conducting-like screening solvation model (COSMO)[19] was applied with $\varepsilon = 36.6$ D as appropriate dielectric constant for acetonitrile. In addition, effective core potentials (ECP) were used for Ir[20]. Excited state calculations were facilitated by using the lowest lying triplet, since the excited state triplet and first excited singlet potential energy surfaces are known to be very similar[11]. For all calculations the molecular structure was optimized from crystal structures after which Ir-Ir distance and ligand dihedral twist were constrained. For the ground state calculations, Ir-Ir distance and dihedral twist were constrained to the results of our previous study[10]. For the excited state calculations a total of 121 structures were optimized where the Ir-Ir distance and dihedral twist were systematically varied from 2.5 to 5.0 Å in 11 steps and from 0 to 50 degrees in 11 steps respectively.

Computational Details – BOMD

The acetonitrile (ACN) solvent shell response of solvated complex has been investigated using DFT-based Quantum Mechanical / Molecular Mechanics Born-Oppenheimer Molecular Dynamics (BOMD) simulations, as described in[11], where the results are also compared to previous experimental results, showing good agreement.

Supplementary Figure 14 shows the Ir-solvent pairwise radial distribution functions for 40 ES trajectories binned in 50 fs intervals, and samples the RDF in each time step, resulting in a total of 200 frames used for each ES curve, whereas the GS RDF was sampled over the entire GS trajectory.

In the ground state, the RDF of the ACN methyl groups and the Ir atoms shows a peak around 3.5 Å (Supplenetary Figure 14a). Since this peak has a value of $g(r) < 1.0$, it is still less likely to find a solvent molecule in this region, compared to the bulk solvent. However, since the probability is still higher in this region than in the adjacent space, this peak represents what can be termed a quasi-ordering of the methyl ends with respect to the solvent-accessible Ir-regions of $Ir_2(dimen)_4^{2+}$, parallel to the Ir-Ir axis,

There is no analogue to the Ir-Me peak in the Ir-N RDF. For the C_{ligand} RDFs we observe a steeper increase in probability of finding ACN nitrogen atoms, than methyl groups. These observations correspond to the ACN molecules in the $Ir_2(dimen)_4^{2+}$ side-regions having a preferential orientation

perpendicular to the Ir-Ir axis, with the N ends oriented towards the ligands, and the Me ends towards the metals. This interpretation is supported by a Bader analysis[21] of a snapshot of the GS trajectory, which assigns roughly half a formal positive charge to each Ir atom, approx. 1 negative formal charge to each of the ligand nitrogen atoms, which are then stabilized by the electro-positive methyl ACN ends.

For the excited state distributions we observe a fast (< 1 ps) decay and ~ 0.7 Å displacement of the $g_{\text{Ir-Me}}(r)$ peak, and an equally fast grow-in of the peak around $r = 9$ Å. On longer time scales (> 2 ps), a clear peak in the $g_{\text{Ir-N}}(r)$ starts to grow in at around 3.5 Å.

The t < 1 ps features are interpreted as arising from the metal atoms contracting, but as seen from the perspective of the solvent, and are thus not related to the solvent cage itself undergoing significant dynamics.

The model of the (changes in) quasi-ordering of the solvent is further underpinned by Supplementary Figure 15. The figure shows the coordination number ratios of Ir-N:Ir-Me (**a**), and Ligand-N:Ligand-Me (**b**). A value of 1 is equivalent to a random orientation, since there is an equal amount of nitrogen atoms and methyl groups in ACN. In the GS, the Ir ratio at shorter distances is below 1, meaning that the solvent has a preferred orientation of pointing the nitrogen atoms away from the Ir atoms. This changes significantly in the excited state trajectories, meaning that the preferred orientation inverts after excitation such that the Ir atoms are now preferably solvated by the nitrogen atoms of the acetonitrile.

With time, the ACN-rotation shown in Supplementary Figure 14 settles, resulting in an Ir-N peak growing in around $r \sim 3$ Å (Supplementary Figure 14). The temporal evolution is visualized in Supplementary Figure 16, which is a 2D plot of cut-outs of the relevant *r* regions, evolving in time. In the top plot, the Ir-Ir contraction is again evident. At $t > 2$ ps, the Ir-N peak starts to grow in upon coordination of the nitrogen groups of the acetonitrile solvent.

Supplementary References:

1. Hartsock, R. W., Zhang, W., Hill, M. G., Sabat, B., Gaffney, K. J. Characterizing the Deformational Isomers of Bimetallic $Ir_2(dimen)_4^{2+}$ (dimen = 1,8-diisocyano-p-menthane) with Vibrational Wavepacket Dynamics, *J. Phys. Chem. A*, **115**, 2920-2926 (2011).
2. Harmand, M. *et al.* Achieving few-femtosecond time-sorting at hard X-ray free-electron lasers. *Nature Photonics*, **7,** 215–218, (2013).
3. van Driel, T. B. *et al.* Disentangling detector data in XFEL studies of temporally resolved solution state chemistry, *Faraday discussions*, **117**, 443-465 (2015).
4. Hura, G. *et al.* A high-quality x-ray scattering experiment on liquid water at ambient conditions. *The Journal of Chemical Physics*, ***113***(20), 9140 (2000)
5. Pauw, B. R. Everything SAXS: small-angle scattering pattern collection and correction, *Journal of Physics: Condensed Matter* ***25***, 383201(2013).
6. Haldrup, K., Christensen, M., Nielsen, M. M. Analysis of time-resolved X-ray scattering data from solution-state systems, *Acta Cryst. A*, **66**, 261–269 (2010).
7. Christensen, M. *et al.* Time-Resolved X-ray Scattering of an Electronically Excited State in Solution. Structure of the $^3A_{2u}$ State of Tetrakis-μ-pyrophosphitodiplatinate(II), J. Am. Chem. Soc., **131**, 502-508, (2009).

8. Lorenz, U., Møller, K. B., Henriksen, N. E. On the interpretation of time-resolved anisotropic diffraction patterns. *New Journal of Physics*, ***12***(11), 113022 (2010).
9. Kim, J. *et al.* Anisotropic Picosecond X-ray Solution Scattering from Photoselectively Aligned Protein Molecules, *The Journal Of Physical Chemistry Letters*, 350-356, (2011)
10. Haldrup K. *et al*. Bond shortening (1.4 Å) in the singlet and triplet excited states of $[Ir_2(dimen)_4]^{2+}$ in solution determined by time-resolved X-ray scattering, *Inorg. Chem*., **50**, 9329-9336 (2011).
11. Dohn, A. *et al.* Direct dynamics studies of a binuclear metal complex in solution: the interplay between vibrational relaxation, coherence, and solvent effects, *J. Phys. Chem. Lett.*, **5**, 2414-2418 (2014).
12. Kjaer, K. S. *et al*. Introducing a standard method for experimental determination of the solvent response in laser pump, X-ray probe time-resolved wide-angle X-ray scattering experiments on systems in solution, *Phys. Chem. Chem. Phys*., **15**, 15003-15016 (2013).
13. Canton, S. *et al.* Visualizing the non-equilibrium dynamics of photoinduced intramolecular electron transfer with femtosecond X-ray pulses, *Nature Commun.*, **6**, 6359 (2015).
14. Cammarata, M. *et al.* Impulsive solvent heating probed by picosecond x-ray diffraction., *J. Chem. Phys.* **124**, 124504 (2006).
15. Dohn, A. O., Biasin, E., Haldrup, K., Nielsen, M.M., Henriksen, N.E., Møller, K.B. On the calculation of x-ray scattering signals from pairwise radial distribution functions, *J. Phys. B: At. Mol. Opt. Phys*, accepted for publication (2015)
16. Neese, F. The ORCA program system, *WIREs Comput. Mol. Sci.* **2**, 73–78 (2012).
17. Adamo, C. *et al.* Toward reliable density functional methods without adjustable parameters: The PBE0 model, *The Journal of Chemical Physics* **110**, 6158-6170 (1999).
18. Weigend, F. *et al.* Balanced basis sets of split valence, triple zeta valence and quadruple zeta valence quality for H to Rn: Design and assessment of accuracy, *Phys. Chem., Chem. Phys.***7**,3297-3305 (2005).
19. Klamt, A. *et al.* COSMO: a new approach to dielectric screening in solvents with explicit expressions for the screening energy and its gradient, *J. Chem. Soc., Perkin Trans.* 2 **5**,799-805 (1993)
20. Andrae, D. *et al.* Energy-adjusted ab initio pseudopotentials for the second and third row transition elements, *Theor. Chim. Acta 77*, **123** (1990).
21. Tang, W. *et al.* A Grid-Based Bader Analysis Algorithm Without Lattice Bias. *Journal of Physics: Condensed Matter*, 084204 (2009).
